# Elasticity Approach to Predict Shape Transformation of Functionally Graded Mechanical Metamaterial under Tension

**DOI:** 10.3390/ma14133452

**Published:** 2021-06-22

**Authors:** Mohammad Javad Khoshgoftar, Ali Barkhordari, Sajjad Seifoori, Mohammad Javad Mirzaali

**Affiliations:** 1 Department of Mechanical Engineering, Faculty of Engineering, Arak University, Arak 38156-88349, Iran; ali.barkhordarii95@gmail.com; 2 Department of Mechanical Engineering, Faculty of Engineering, Vali-e-Asr University of Rafsanjan, Rafsanjan 77188-97111, Iran; sajjad.seifoori@vru.ac.ir; 3 Department of Biomechanical Engineering, Faculty of Mechanical, Maritime, and Materials Engineering, Delft University of Technology (TU Delft), Mekelweg 2, 2628 CD Delft, The Netherlands

**Keywords:** mechanical metamaterials, auxetic, re-entrant structures, finite element modeling, theory of elasticity, shape matching

## Abstract

The re-entrant structures are among the simple unit cell designs that have been widely used in the design of mechanical metamaterials. Changing the geometrical parameters of these unit cell structures, their overall elastic properties (i.e., elastic stiffness and Poisson’s ratio), can be simultaneously tuned. Therefore, different design strategies (e.g., functional gradient) can be implemented to design advanced engineering materials with unusual properties. Here, using the theory of elasticity and finite element modeling, we propose a fast and direct approach to effectively design the microarchitectures of mechanical metamaterials with re-entrant structures that allow predicting complex deformation shapes under uniaxial tensile loading. We also analyze the efficiency of this method by back calculating the microarchitectural designs of mechanical metamaterials to predict the complex 1-D external contour of objects (e.g., vase and foot). The proposed approach has several applications in creating programmable mechanical metamaterials with shape matching properties for exoskeletal and soft robotic devices.

## 1. Introduction

Cellular materials (e.g., bone, wood, and cork) can be extensively found in nature. These natural cellular materials have been sources of inspiration for engineers for many decades to create bioinspired lattice structures [1], for instance, those with low density and high stiffness [2] or high energy absorption properties [3]. Design motifs, such as hierarchy and functional gradient, are examples of design principles that exist broadly in natural materials and have been widely used to develop bio-inspired cellular structures [4,5]. The re-entrant unit cells are among those cellular microstructures that found their roots in nature and have been utilized to design advanced engineering materials, such as designer materials or mechanical metamaterials [6,7] (i.e., materials whose mechanical properties originate directly from their architectural designs at a lower scale and not from their chemical compositions).

The 2D mechanical metamaterials made from re-entrant structures are composed of hexagonal repetitive unit cells arranged rationally in a plane. As re-entrant unit cells belong to the class of bending-dominated [8,9] unit cells, their particular strut’s orientation can result in unusual properties, such as negative Poisson’s ratio (i.e., auxetic [10]), when stretched or compressed in-plane or tunable (i.e., synclastic, anticlastic) curvatures when bent out-of-plane [11,12].

One of the unique features in the design of re-entrant lattices is their inherent simplicity. This means a relatively low number of design parameters are necessary to design re-entrant lattices. That is probably one reason why these particular unit cell designs have received a great deal of attention with respect to other types of unit cell designs (e.g., chiral, rigid rotational structures, and crumpled and perforated sheet models [13]). For example, only by changing the angle of re-entrant unit cells, one can achieve a wide range of elastic properties (i.e., elastic stiffness and positive or negative values of Poisson’s ratio) [14]. When this simple geometry is combined with the concept of multi-materials, it can provide even additional freedom for the designer to boost their properties as compared to those of monolithic lattice structures [15].

Furthermore, by a rational combination of re-entrant unit cells with different values of Poisson’s ratios, one can control the local (e.g., action-at-a-distance [16]) or global deformation (e.g., shape-matching mechanical metamaterials [17]) of the lattice structures under uniaxial far-field loading. This can then lead to the design of programmable mechanical materials with shape morphing or shape transformation properties with numerous applications in the fields of architecture, sports, soft robotics, mechanical and biomedical engineering, and aeronautical industries [18,19,20,21,22,23,24].

The state-of-the-art design of programmable mechanical materials with shape morphing and shape transformation properties requires spatial combinations of various unit cells to guide the deformation patterns within the structure and avoid any unnecessary strain localizations. This makes the design of such programmable metamaterials using re-entrant structures more challenging. It is because the re-entrant lattices exhibit anisotropic properties, which means their properties are different in two principal directions. Although several analytical relations have been proposed to determine the elastic properties of re-entrant structures [25,26,27,28,29,30], these models cannot predict their shape transformation properties, particularly when more complex microarchitectural designs (e.g., functional gradient) have been used. Computational modeling (e.g., finite element (FE) models) of re-entrant structures has provided a powerful tool in predicting their design envelop [31,32,33,34,35,36]. However, again in the case of complex design, these models will often become computationally expensive.

Here, using the theory of elasticity and finite element modeling, we propose a methodology to predict the deformation patterns in mechanical metamaterials created from re-entrant building blocks. The method offers a high-speed and direct approach in first, predicting their elastic properties and second, back calculating their microarchitectural designs. We also analyzed the efficiency of this approach in creating the programmable mechanical metamaterials with even more complex (e.g., functionally graded) microstructures by comparing our results with those of the literature. The proposed method has several applications in designing programmable mechanical metamaterials with shape matching properties for exoskeletal and soft robotic devices.

## 2. Modeling Approach

**Figure 1:**
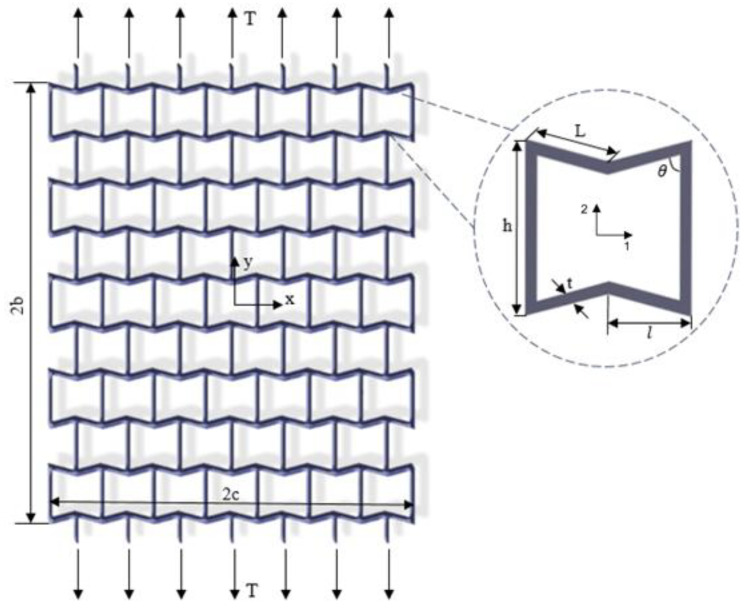
Re-entrant lattice structure is made of hexgonal unit cells whose geometrical parameters are shown in the subfigure. The lattice structure is supposed to be under uniaxial tensile loading.

We used the conventional 2D re-entrant unit cell structural design proposed by Gibson et al. [37] in this study. The re-entrant structure was assumed to be subjected to uniaxial tensile load T along the *y*-axis (Figure 1). Cell length (L), cell height (h), cell angle (θ), and cell thickness (t) are geometrical parameters of the re-entrant unit cell structure (see Figure 1). First, we modeled re-entrant designs using the classical 2D theory of elasticity (i.e., Airy function [38]). Since the structure thickness was assumed to be small compared to the plate dimensions, plane stress equations were used for these analyses. This means that the in-plane shear strain and shear stress were assumed to be zero. We compared our results with finite element modeling (see Section 2.2) to validate our analytical approach.

### 2.1. Elasticity Modeling

The 2D elasticity theory for a thin plate regardless of body force leads to the biharmonic equation (∇^4 φ = 0), where φ is an Airy stress function. Stress fields derived from an Airy stress function satisfy the equilibrium equation and correspond to compatible strain fields. Airy stress function is suitable to solve stress boundary condition problems. The only component of the stress boundary condition here is the in-plane tensile loading applied along the *y*-direction (Figure 1). Therefore, other stress components (i.e., $\sigma_{xx}$,$\tau_{xy}$) could be excluded, and the Airy stress function is obtained as follows:

(1) $$\varphi =Ax^{2}$$

The re-entrant structure is under stretch loading, as mentioned above and shown in Figure 1. The stress boundary conditions are written in Equation (2), where $\sigma_{xx}$ and $\sigma_{yy}$ are the normal stresses in the *x* and *y* direction, and $\tau_{xy}$ is the in-plane shear stress.

(2) $$\{\begin{array}{c} \sigma_{xx}\left(\pm c,y\right)=0\\ \sigma_{yy}\left(x,\pm b\right)=\pm T\\ \tau_{xy}\left(\pm c,y\right)=\tau_{xy}\left(x,\pm b\right)=0 \end{array}$$

The stress field can be calculated according to Airy functions as Equation (3).

(3) $$\sigma_{xx}=\frac{\partial^{2}\varphi }{\partial^{2}y};\sigma_{yy}=\frac{\partial^{2}\varphi }{\partial^{2}x};\tau_{xy}=\frac{\partial^{2}\varphi }{\partial x\partial y}$$

The unknown coefficient (A) of the Airy function can be obtained from boundary conditions shown in Equation (2) as follows:

(4) $$A=\frac{T}{2}$$

The stress–strain relationship according to Hooke’s law for orthotropic re-entrant structure under an in-plane state of stresses can be calculated as:

(5) $$\left[\begin{array}{c} \sigma_{xx}\\ \sigma_{yy}\\ \tau_{xy} \end{array}\right]=\left[\begin{array}{ccc} c_{11} & c_{12} & 0\\ c_{21} & c_{22} & 0\\ 0 & 0 & c_{66} \end{array}\right]\left[\begin{array}{c} \varepsilon_{xx}\\ \varepsilon_{yy}\\ \gamma_{xy} \end{array}\right]$$

where $c_{ij}$ are elastic constants that can be obtained from Equation (6).

(6) $$c_{11}=\frac{E_{11}}{1-\upsilon_{12}\upsilon_{21}},c_{12}=\frac{E_{22}\upsilon_{12}}{1-\upsilon_{12}\upsilon_{21}}\;c_{22}=\frac{E_{22}}{1-\upsilon_{12}\upsilon_{21}},c_{66}=G_{12}$$

where $G_{12}$, $E_{11}$, $E_{22}$, $v_{12}$, and $v_{21}$ are respectively the shear modulus, Young’s moduli, and Poisson’s ratios in principal directions (Figure 1). The mechanical properties, according to the unit cell geometrical parameters shown in Figure 1, can be written as follows [39]:

(7) $$\begin{array}{l} L=\frac{l}{\sin\theta }\\ \upsilon_{12}=-\frac{\cos\theta \left(1-{\left(\frac{t}{L}\right)}^{2}\right)\left(\frac{h}{L}-\cos\theta \right)}{\left(\sin^{2}\theta \right)\left(1+\left(\left(\cot^{2}\theta +\csc^{2}\theta \right)\left(\frac{h}{L}\right)\right){\left(\frac{t}{L}\right)}^{2}\right)}\\ \upsilon_{21}=-\frac{\cos\theta \left(1-{\left(\frac{t}{L}\right)}^{2}\right)}{\left(\cot^{2}\theta +{\left(\frac{t}{L}\right)}^{2}\right)\left(\frac{h}{L}-\cos\theta \right)}\\ E_{11}=E{\left(\frac{t}{L}\right)}^{3}\frac{\left(\frac{h}{L}-\cos\theta \right)}{\left(1+\left(\cot^{2}\theta +\frac{h}{L}\csc^{2}\theta \right){\left(\frac{t}{L}\right)}^{2}\right)\sin^{3}\csc^{2}\theta }\\ E_{22}=E{\left(\frac{t}{L}\right)}^{3}\frac{1}{\sin\theta \left(\frac{h}{L}-\cos\theta \right)\left(\cot^{2}\theta +{\left(\frac{t}{L}\right)}^{2}\right)} \end{array}\;G_{12}=E{\left(\frac{t}{L}\right)}^{3}\frac{1}{\left(\frac{h}{L}\right)\left(1+2\frac{h}{L}\right)\cos\theta }$$

The strain relations (Equation (8)) can be calculated by replacing the Airy stress functions (Equation (3)) in Equation (5).

(8) $$\begin{array}{r} \varepsilon_{xx}=-\frac{T\upsilon_{12}\left(-1+\upsilon_{12}\upsilon_{21}\right)}{-E_{11}+E_{22}\upsilon_{12}{}^{2}}\\ \varepsilon_{yy}=-\frac{T\left(-1+\upsilon_{12}\upsilon_{21}\right)}{E_{11}-E_{22}\upsilon_{12}{}^{2}}\\ \gamma_{xy}=0 \end{array}$$

By integrating Equation (8), the displacement field in the *x* and *y* directions (i.e., u and v) can be determined as:

(9) $$u=-\frac{T\upsilon_{12}\left(-1+\upsilon_{12}\upsilon_{21}\right)}{-E_{11}+E_{22}\upsilon_{12}{}^{2}}x+g\left(y\right)\;v=-\frac{T\left(-1+\upsilon_{12}\upsilon_{21}\right)}{E_{11}-E_{22}\upsilon_{12}{}^{2}}y+f\left(x\right)$$

The functions f and g are unknown functions that can be calculated by applying the shear strain relation given in Equation (8):

(10) $$\frac{\partial u}{\partial y}+\frac{\partial v}{\partial x}=2\gamma_{xy}=0\to \overset{´}{f}\left(x\right)=-\overset{´}{g}\left(y\right)=constant$$

By integrating from Equation (10), the unknown functions can be written as:

(11) $$f\left(x\right)=wx+v_{0}\;g\left(y\right)=-wy+u_{0}$$

where $u_{0}$, $v_{0}$, and w are arbitrary constants of integration that indicate the translation and rotation of the plate. Since the structure is symmetrical, regardless of the rotation and rigid displacements, the displacement of the plate is expressed using Equations (12) and (13).

(12) $$u=-\frac{T\upsilon_{12}\left(-1+\upsilon_{12}\upsilon_{21}\right)}{-E_{11}+E_{22}\upsilon_{12}{}^{2}}x$$

(13) $$v=-\frac{T\left(-1+\upsilon_{12}\upsilon_{21}\right)}{E_{11}-E_{22}\upsilon_{12}{}^{2}}y$$

### 2.2. Finite Element Modeling

**Figure 2:**
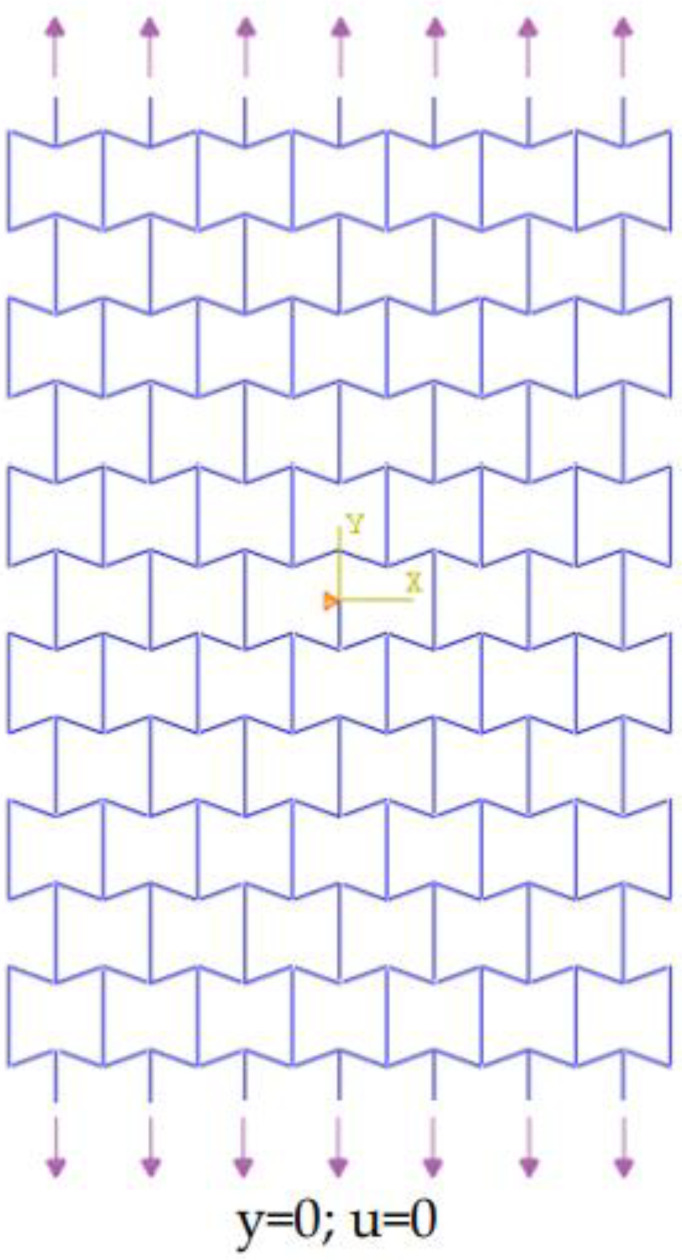
Finite element model and boundary conditions.

Finite element modeling (FEM) of the re-entrant structure has been performed by Abaqus 6.12.1, as shown in Figure 2. The 2D beam element (B31, linear element based on Timoshenko formulation) is used for computational simulation, which has a low analysis run time. We performed a mesh sensitivity analysis that showed the displacement results converged by element size equal to L/10. Because of the symmetry of the plate, the midpoint of the plate was constrained in the *x*-direction. The upper and lower edges of the plate were in tension. The equivalent load in the *y*-direction must be equal to the corresponding load in the elasticity theory. Python programming language was used to parametrically model the structure in Abaqus.

## 3. Results and Design Producer

**Figure 3:**
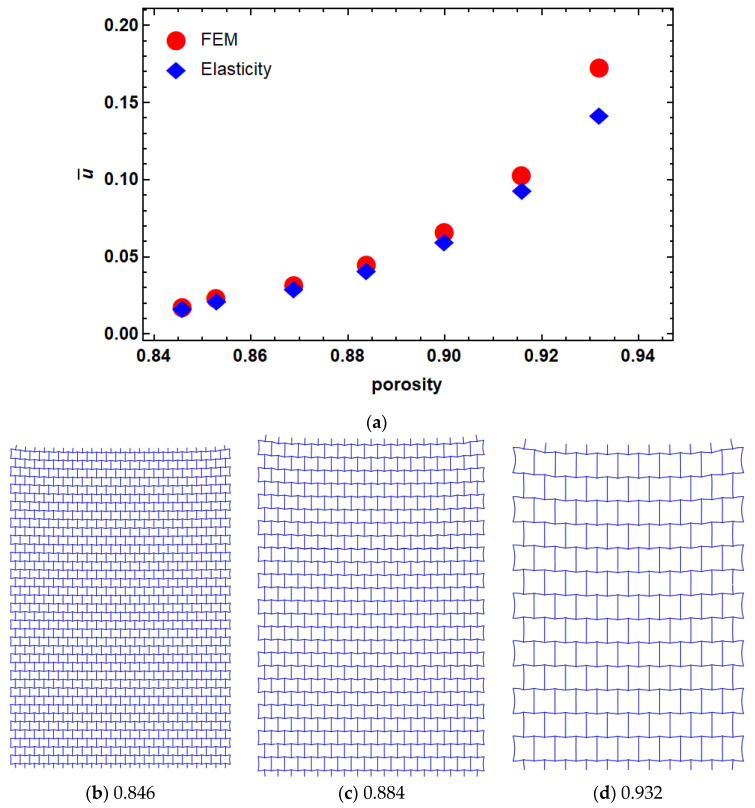
Dimensionless displacement of re-entrant structure with different porosities (**a**) porosity. Examples of different porosity levels are shown subfigures (**b**) 0.846, (**c**) 0.884 and (**d**) 0.932.

First, the effects of porosity on the elasticity solution were studied. The area of the lattice structure was directly calculated from Abaqus. We then calculated the void space by subtracting this area from the equivalent solid plate. The porosity was defined as the ratio of the void space filling to the equivalent solid plate. The porosity value can therefore change between 0 and 1, where 0 means the fully solid plate. Here, we changed the porosity of the lattice structures by changing the dimensions of their unit cells (i.e., h and L) while the strut thickness (i.e., t) and unit cell angle (i.e., θ) were kept unchanged (see Figure 3b–d).

The finite element model was compared with the results obtained from the elasticity approach with different porosities in Figure 3. The dimensionless results are represented as $\bar{u}=\frac{u\left(x=c,y\right),\bar{E}}{2bT}10^{-3}$, where $\bar{E}$ is the mean values of the elastic modulus of the lattice structure. As it can be seen in Figure 3, there is a good agreement between FEM and the results of the elasticity approach at a lower level of porosities. However, by increasing the porosity, the difference between the two models increased. This can be due to the rotation of unit cells in higher porosities that can lead to in-plane shear strain, thus violating our assumption regarding the zero-shear strain condition.

### 3.1. Variable Porosity Modeling

More complex re-entrant structures were modeled by changing their cell parameters in a way to have functionally graded unit cells in the *y*-direction and repeated unit cells in the *x*-direction. Since the unit cell angle has the greatest effect on the Poisson’s ratio [14], the presented model has an angle varying in the longitudinal direction. To satisfy the compatibility with other unit cell designs, the unit cell height also changed in each row according to a variable unit cell angle. The structure was divided into several rows with a specific angle in the longitudinal direction. As an example, a model with a linear variable angle was created as Equation (14).

(14) θ=48+0.7461y

**Figure 4:**
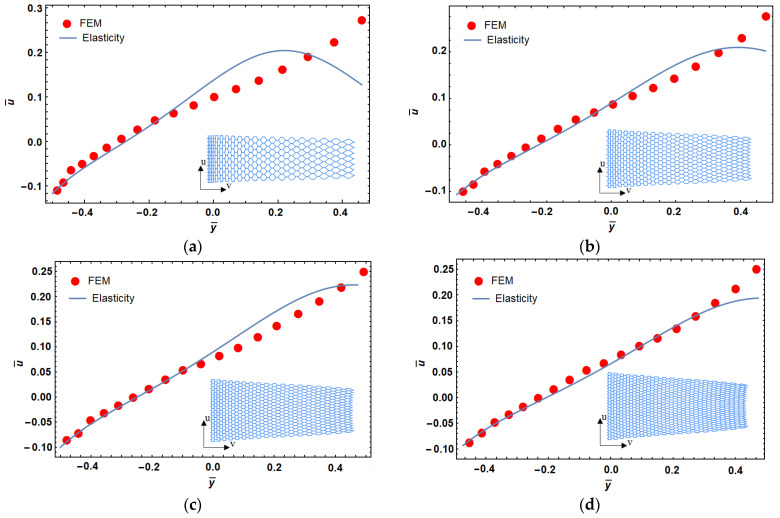
Porosity effect on dimensionless displacement in functionally graded reentrant structure; 94% (**a**), 92% (**b**), 90% (**c**), and 88% (**d**) porosity.

The mechanical properties of the structure are obtained by replacing Equation (14) with Equation (7). Although the material properties vary along the *y*-direction, the presented elasticity solution remains valid because the stress function does not depend on y. Therefore, a variable deformation along the *y*-direction is the result of heterogeneity of mechanical properties in the *y*-direction. The dimensionless deformation ($\bar{u}$) vs. normalized length ($\bar{y}=\frac{y}{b}$) of a lattice structure with different levels of porosity are shown in Figure 4, where $\frac{b}{c}=2.5$ and $\frac{h}{l}=2$. It can be seen in this figure that the difference between these two models (i.e., FEM and elasticity approach) increased by increasing the porosity. As mentioned above, this can be explained by creation of shear strain due to cell rotations in higher porosities. Based on this observation, we fixed the level of porosity to ~90% for our lattice designs hereafter.

**Figure 5:**
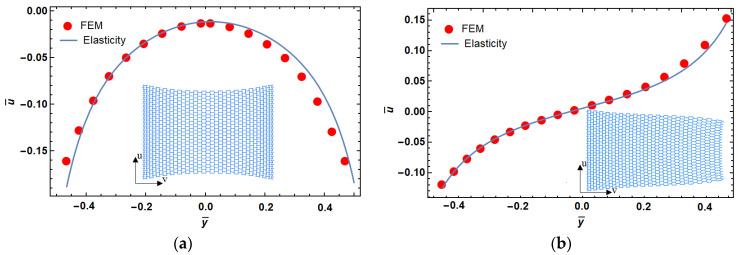
Dimensionless displacement in functionally graded re-entrant structure for the desired parabola (**a**) and cubic (**b**) shapes.

Two other models with higher polynomial order for unit cell angle in a function of *y*-coordinate (rows) were considered in Figure 5 with the porosity of 88%. Equations (15) and (16) show the change of angle in the longitudinal direction for these models. These models consist of 23 × 35 cells and the dimension ratio with $\frac{b}{c}=2.5$ and $\frac{h}{l}=2$. As it can be seen in Figure 5, the deformed structure has an arbitrary shape that can be used for reaching a specific design that will be discussed in the next section.

(15) $$\theta =42.0628+1.679y-0.0161y^{2}$$

(16) *θ* = 51.838 + 1.16*y* − 0.01202*y*^2^ + 0.0000644*y*^3^

### 3.2. Variable Porosity Designing

In this section, a method is presented to design a functionally graded re-entrant structure. Programming the microstructure to reach the desired deformation in tension is the main goal of this section. For this purpose, the angle of unit cells of the re-entrant structure must be calculated in terms of displacement.

**Figure 6:**
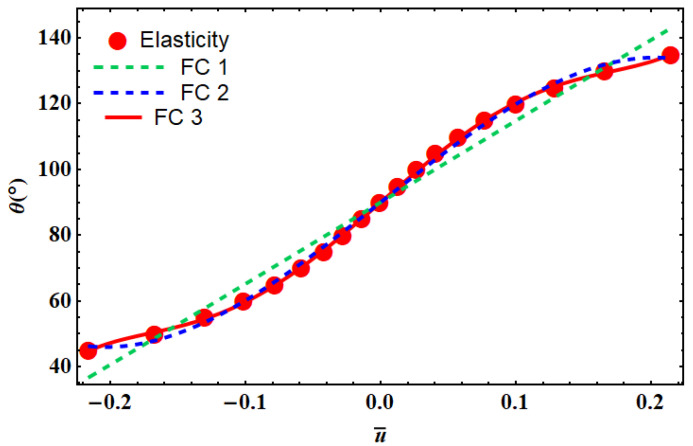
Different fitting curves (FC) used to find the relationship between the unit cell angle vs. the dimensionless lateral displacements using the elasticity theory. The corresponding fitting equations are shown in Table 1.

The angle effect on the displacement field can be obtained from Equation (12). The angle of the unit cell affects the mechanical properties of the structure and can change the displacement field. Therefore, the displacement field was assumed to be a function of the angle of unit cells, as shown in Figure 6. We fitted a curve to calculate the angle of the unit cell as a function of displacement. Three different fitting curves were used (Table 1) that were calculated with MATLAB (R2014a, MathWorks, Natick, MA, USA). There is a minimum error for FC3 (Fit Curve 3) that consisted of polynomial and trigonometric functions (Table 1).

The desired deformation in tensile loading can be obtained by a re-entrant cellular structure using variation in angle. This change has the strongest effect on the mechanical properties of the lattice structure. The angle of each row can be obtained by fitting equations introduced in Table 1.

The angle of each row is dependent on the desired displacement. The desired displacement of each row must be calculated in the middle of the ligament of each row. The location of this point can be calculated from Equation (17), where *m* is the number of segments and *n* is the row number.

(17) $$y=\overset{m}{\underset{n=1}{\sum }}h_{n}=\overset{m}{\underset{n=1}{\sum }}\left(\frac{b}{m}-Lsin\left(\theta_{n}\right)\right)$$

To show the capability of this method, a typical linear deformation is considered as Equation (18).

(18) u=−0.19577+0.00288y

**Figure 7:**
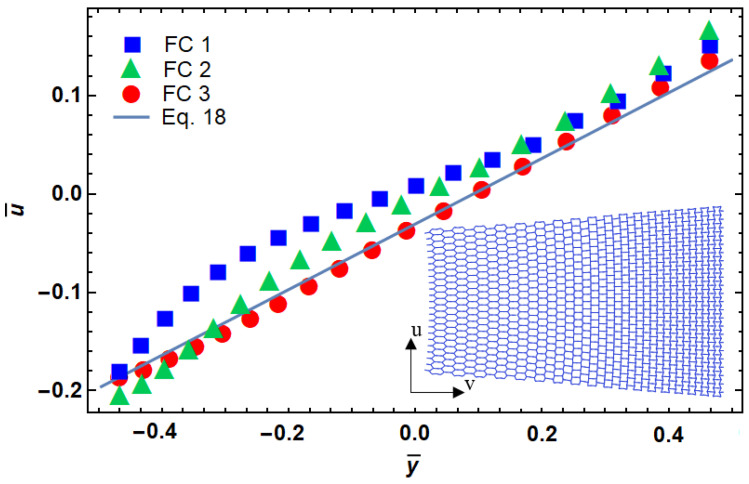
Functionally graded design of a re-entrant cellular plate with non-dimensional lateral and longitudinal deformation.

By dividing the height of the structure into 35 rows, the angle of each cell can be back-calculated from Table 1 and Equation (18). Figure 7 shows a comparison of the desired displacement with a designed model that was used in the development of FEM. First, the angle of each row is obtained, and then FEM is performed to see the deformation result. The design based on three different fitting curves, introduced in Table 1, was compared in this figure. It can be seen that FC3 is the best match with the target shape of deformation.

**Figure 8:**
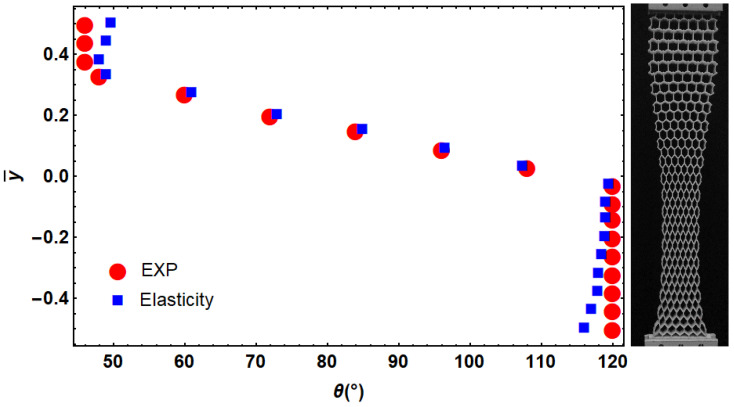
Comparison between the designed angle of cells with the presented approach and those obtained from experimental results presented in [17] for the desired deformation.

The implemented method was compared with experimental test data published in [17]. The deformation field reported in this reference was used to design a functionally graded re-entrant structure with the presented method. The angle of cells was compared by reported angles in Figure 8. We observed a good match between the two approaches. A deviation between two results was observed at regions close to the gripping fixture. The difference in applied boundary conditions can be one of the sources of such difference between two approaches.

**Figure 9:**
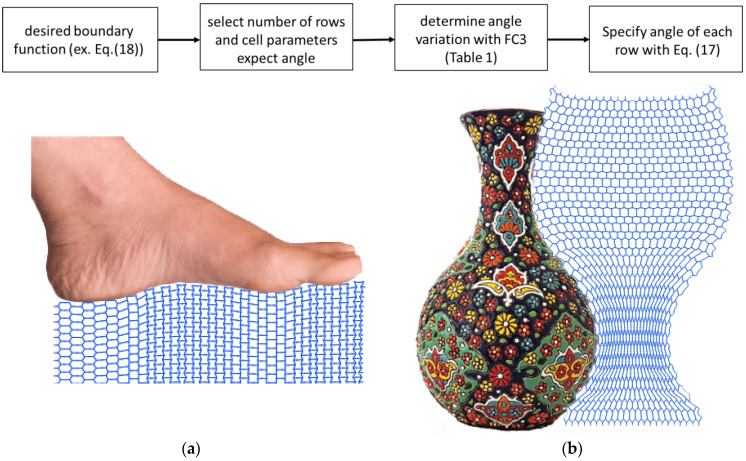
A flowchart is shown in the top row showing the steps necessary for the design of mechanical metamaterials. This flowchart was used to back-calculate the microarchitecture of lattice structures for predicting the complex 1D shapes of two arbitrary examples, namely foot (**a**) and vase (**b**). Based on the elasticity approach proposed here, the microarchitectures of the lattice can be determined using re-entrant unit cells.

To show the application of this method for the design of programmable shape-matching mechanical metamaterials, two arbitrary shapes were used in Figure 9. The first model is a vase structure with a special 1D curved contour line and the second model was the complex shape of a foot. We used our approach to design the corresponding microarchitectures of lattice structures so that under applied tensile loading, a particular 1D curved shape can be achieved. As a consequence, the presented design approach has a wide range of applications in the design of wearable medical (e.g., exo-skeletal prosthesis) and soft robotic devices (e.g., soft grippers to grip delicate objects). Re-entrant cell parameters can be designed with an elasticity approach, instead of other time-consuming methods, such FEM with 2D plane elements. Moreover, the proposed elasticity approach is sensitive to the level of porosity of the final structure as well as the applied boundary condition. Nevertheless, this approach may provide a fast and accurate design tool for the design of shape-matching mechanical metamaterials.

## 4. Conclusions

To conclude, here, the functionally graded mechanical metamaterials made from re-entrant structures under uniaxial tensile load were modeled using the elasticity theory and FEM. The effects of different morphological and geometrical parameters (i.e., porosity) on the overall shape of deformation of such materials were analyzed. We also showed how the presented approach can be used for the back-calculation of the microarchitectural designs of lattice structures in order to achieve any arbitrary target shape of deformation. This approach can be considered an effective tool in the design of programable mechanical metamaterials with shape matching properties. We expect this approach can be extended to the design of 3D structures with the same properties and predicting arbitrary shapes in 2D or 3D. The proposed design method can be used in many applications, such as soft robotics, fashion industries, and medical devices.

## Figures and Tables

**Table 1:** Fitting curves (FC) and corresponding errors.

Fit Curve	Fitting Equation	R^2^
**FC1**	*θ* = 90 + 248.8Sin*u*	0.9722
**FC2**	*θ* = 90 + 325.1*u* − 2621.4*u*^3^	0.9977
**FC3**	*θ* = 90 – 7.5 × 10^6^*u* + 1.24 × 10^6^*u*^3^ + 7.5 × 10^6^Sin*u*	0.9997

## Data Availability

The data presented in this study are available on reasonable request from the corresponding author.
